# Intramuscular dendritic fibromyxolipoma in a 24-year-old male: A case report and review of the literature

**DOI:** 10.3892/ol.2014.2794

**Published:** 2014-12-12

**Authors:** XIA XU, WEN XIONG, LIDUAN ZHENG, JIE YU

**Affiliations:** 1 Department of Pathology, Union Hospital, Tongji Medical College, Huazhong University of Science and Technology, Wuhan, Hubei 430022, P.R. China; 2 Department of Orthopaedics, Wuhan Puai’s Hospital, Wuhan, Hubei 430032, P.R. China; 3 Department of Radiology, Union Hospital, Tongji Medical College, Huazhong University of Science and Technology, Wuhan, Hubei 430022, P.R. China

**Keywords:** intramuscular, dendritic fibromyxolipoma, myxoid spindle cell lipoma, solitary fibrous tumor

## Abstract

Dendritic fibromyxolipoma (DFML) is an uncommon, benign soft tumor that usually arises in the subcutis. To date, ~24 cases of DFML have been reported in the literature and only one of these has been in the muscle. The present study reports the case of a 24-year-old male with a slow-growing, painless mass located deep in the triceps brachii in the left shoulder region. The mass was 14.0×8.5×8.0 cm in size, with well-circumscribed margins. Microscopically, the resected mass was characterized by a proliferation of small spindle or stellate cells, prominent abundant myxoid stroma with ropey collagen bundles and admixed mature adipose tissue. Further immunohistochemical staining indicated that the spindle and stellate cells were reactive with cluster of differentiation 34, vimentin and B-cell lymphoma-2, but not with smooth muscle actin and desmin. Fluorescence *in situ* hybridization showed that the tumor cells did not have the DDIT3 alteration or amplification of MDM2. The tumor was confirmed to be a DFML due to the typical histological, immunophenotypic and genetic findings. To date, subsequent to 4 years of clinical follow-up, there is no sign of recurrence or metastasis. The present study reports a case of DFML in the youngest known patient, and is the second reported case of an intramuscular DFML occurring in the triceps brachii in the left shoulder region. The study discusses the clinicopathological features and the differential diagnosis of DFML, with a review of the literature.

## Introduction

Dendritic fibromyxolipoma (DFML) is an uncommon benign soft-tissue tumor, which is usually located in the subcutis of the head and neck, shoulders, calf, foot, back or groin, with only one previous case reported in the muscle (1–6). DFML often occurs in older males, presenting as a slowly growing mass, which is described as a well-demarcated, encapsulated soft-tissue mass, with a gray-yellow, mucoid or gelatinous cut surface. Microscopically, the classic lesion consists of small spindle or stellate cells, prominent abundant myxoid stroma with ropey collagen bundles and admixtures of mature adipose tissues. A prominent delicate plexiform capillary vascular network is present throughout the tumor. Immunohistochemically, the spindle and stellate cells stain strongly for cluster of differentiation (CD)34, vimentin and B-cell lymphoma (Bcl)-2 (7,8). The adipocytes, but not the spindle or stellate cells, show positive staining for S-100 protein. Staining for keratin, epithelial membrane antigen, smooth muscle actin and desmin is also negative. The present study reports the second known case of intramuscular DFML and reviews the relevant literature. The study was approved by the ethics committee of Union Hospital, Tongji Medical College (Wuhan, China) and written informed consent was obtained from the patient.

## Case report

**Figure 1:**
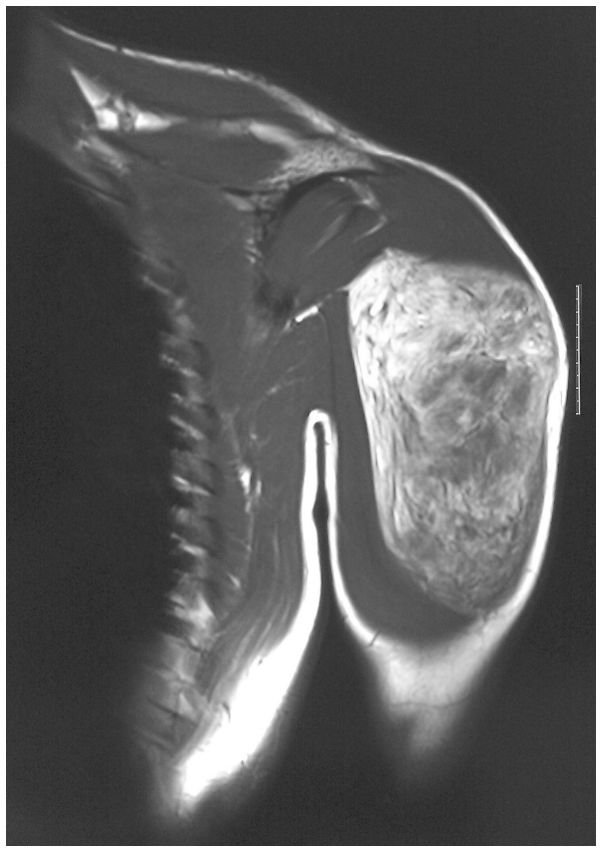
Coronal T1-weighted magnetic resonance imaging of the left shoulder. A large mass is located at the triceps brachii, causing extrusion of the deltoid muscles and teres minor.

A 24-year-old male was admitted to Union Hospital, Tongji Medical College with a painless, slowly growing mass in the left shoulder that had been present for two years. The patient had no previous medical history of disease. Magnetic resonance imaging revealed that the mass was located at the triceps brachii, causing extrusion of the deltoid muscles and teres minor. The mass exhibited well-circumscribed margins and mixed signal intensity (Fig. 1). No other masses or enlarged lymph nodes were present. The patient was successfully treated with a complete resection of the mass. Following the surgery, no further treatment was required.

The excised specimen measured 14.0×8.5×8.0 cm, and was an ovoid, well-encapsulated soft-tissue mass, with a little muscle in the surface. The cut surface was yellow-gray, myxoid and soft, with interspersed gray-white gelatinous-like material. There were no visible areas of necrosis, hemorrhage or cystic degeneration.

**Figure 2:**
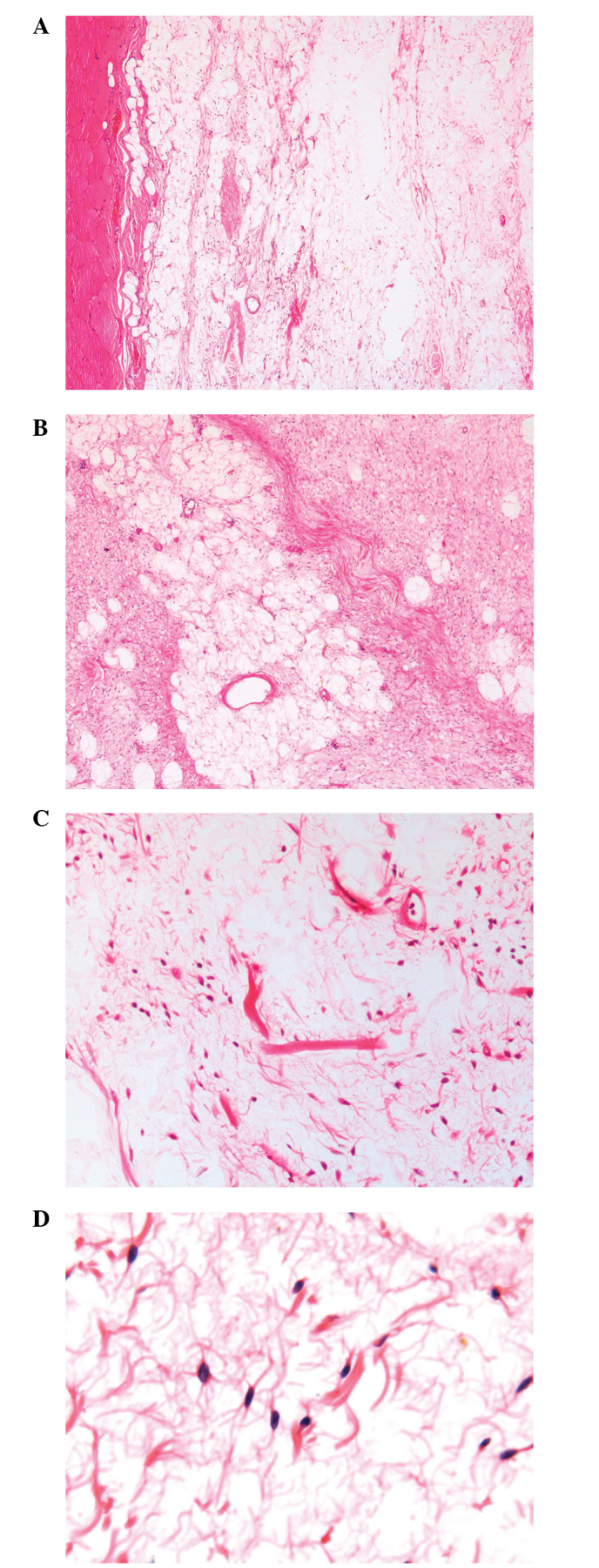
Histopathology of dendritic fibromyxolipoma (DFML). (A) Low-power view showing the tumor cells focally involving skeletal muscle [hematoxylin and eosin (H&E) stain; original magnification, ×40]. (B) The tumor cells and admixed mature adipose tissue (H&E stain; original magnification, ×40). (C) Ropey collagen bundles, which were similar in morphology to solitary fibrous tumor (H&E stain; original magnification, ×200); (D) Spindle cells with dendritic cytoplasmic processes (H&E stain; original magnification, ×400).

Microscopically, the tumor was characterized by a proliferation of small spindle or stellate cells, prominent abundant myxoid stroma with ropey collagen bundles, which were similar in morphology to solitary fibrous tumor (SFT), and an admixture of mature adipose tissues (Fig. 2). The tumor cells were bland without atypical features. Mitotic figures were absent, and the initial diagnosis was of a myxoid liposarcoma.

**Figure 3:**
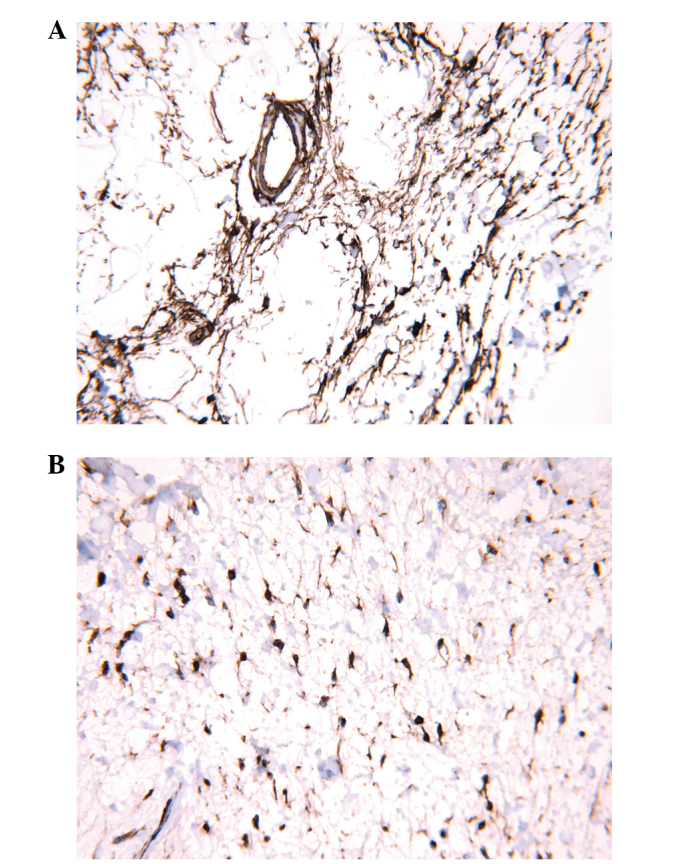
Immunostaining of dendritic fibromyxolipoma. (A) Spindle and stellate cells strongly expressing cluster of differentiation 34 (original magnification, ×400). (B) Spindle and stellate cells strongly expressing vimentin (original magnification, ×400).

The spindle and stellate cells expressed CD34 and vimentin strongly (Fig. 3). They cells were also variably positive for Bcl-2, and negative for smooth muscle actin and desmin. The adipocytes, but not the spindle or stellate cells, expressed S-100 protein. MIB-1 (Ki-67) showed low proliferative activity.

**Figure 4:**
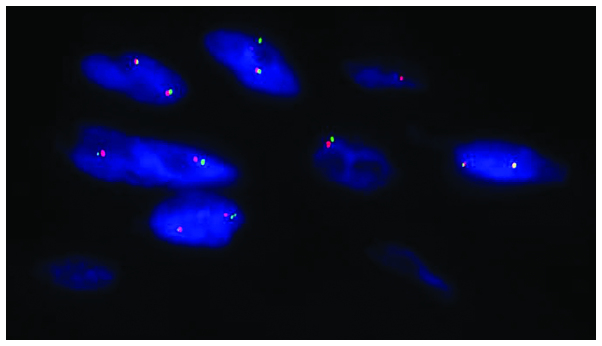
Fluorescence *in situ* hybridization patterns obtained from paraffin-embedded tissue sections, showing a pattern of 2 orange and 2 green signals, without DDIT3 alteration.

The tumor was analyzed by fluorescence *in situ* hybridization (FISH), and revealed the absence of DDIT3 alteration (Fig. 4) or MDM2 amplification, thus supporting the diagnosis of DFML and excluding a diagnosis of myxoid liposarcoma.

To date, there is no sign of recurrence or metastasis subsequent to 4 years of clinical follow-up.

## Discussion

Spindle cell lipoma (SCL) is a benign adipocytic tumor. Several morphological subtypes have been described, including the dermal/cutaneous variant, the angiomatous variant and the fibrous variant. Certain SCLs show extensive myxoid changes and spindle cells with dendritic cytoplasmic processes (7–9). As first described by Suster *et al* in 1998, DFML was distinguished from SCL by the presence of dendritic cytoplasmic processes, a plexiform vascular pattern and abundant keloidal collagen (1). With further study, these features of DFML have also been observed in certain cases of SCL. Karim *et al* (2) speculated that DFML possibly represented an unusual variant of myxoid SCL, due to the similarities in their clinical and pathological features. By contrast, Tan and Wen (3) inferred that DFML occurred on a morphologic continuum and represented a transitional form between spindle cell lipoma and SFT, and SFT was on the end of the spectrum. DFML represents a transitional form between spindle cell lipoma and SFT, and the latter is at the end stage of the transition.

To date, ~24 cases of DFML have been reported in the English or Chinese literature, and cited in PubMed (1–6). A review of these cases shows that the age of the patients ranged between 24 and 81 years, and the median age at diagnosis was 66.5 years. Among the cases, there was a higher prevalence of males; only four cases were reported in females. The majority of tumors were located in the superficial soft tissue of the head and neck region, chest, back, shoulder and lower leg, while only two cases (including the present case) were in the intramuscular location of the shoulder and one case was adherent to the median nerve in the left forearm (5). Intramuscular SCLs can be well circumscribed, with focally involved skeletal muscle. A few cases of infiltrated skeletal muscle, similar to intramuscular lipomas, have also been reported (10–12). In the two reported cases of DFML, that of Karim *et al* and the present study, the pattern of growth belongs to the former, with focally involved skeletal muscle.

In the 24 cases, the masses ranged in size between 2 and 24 cm. The tumors were characterized by a proliferation of small spindle or stellate cells, prominent abundant myxoid stroma with ropey collagen bundles, and an admixture of mature adipose tissues, which is similar to the morphology of SCL. Besides these features, chondroid metaplasia is an uncommon feature of DFML, which has been described by Karim *et al* (2).

In clinical practice, DFML has abundant myxoid stroma and a prominent plexiform vascular pattern reminiscent of that observed in myxoid liposarcoma, therefore, the tumor may be mistaken for a myxoid liposarcoma, which may include myxofibrosarcoma, low-grade fibromyxoid sarcoma and myxoid synovial sarcoma. Suster *et al* described 12 cases of DFML, of which three were initially misdiagnosed as myxoid liposarcoma and one as myxoid malignant fibrous histiocytoma (1). The case reported by Karim *et al* was initially diagnosed as low-grade liposarcoma on an incisional biopsy (2). The present case was also mistaken for myxoid liposarcoma at the preliminary diagnosis. Myxoid liposarcoma often occurs between the ages of 45–60 years, and ~75% of cases develop in the deep muscles of the lower extremities. Well-differentiated myxoid liposarcoma in such locations requires careful consideration. Immunohistochemically, the tumor cells of the overwhelming majority cases stain for S-100 protein, while CD34 staining is negative. In addition, >90% of myxoid liposarcomas present with a reciprocal translocation between chromosomes 12 and 16: t(12;16)(q13; p11) (13,14). The study by Zhang *et al* (15) showed that molecular testing was useful for all deep-seated and larger lipomatous tumors (>15 cm). Using FISH, the absence of DDIT3 alteration or MDM2 amplification was indicated in the present case, thus differentiating the benign DFML from malignant lipomatous tumors.

Myxofibrosarcoma is generally characterized by a significant degree of nuclear pleomorphism and occasional mitotic figures. The particular features are vacuoles of these pseudolipoblasts and a vascular pattern characterized by large curvilinear vessels. Low-grade fibromyxoid sarcoma exhibits myxoid areas and cytologically bland spindle cells. The spindle cells are characterized by whorled or swirling growth patterns. Myxoid chondrosarcomas are composed of small, distinctly eosinophilic cells typically arranged in small clusters, cords or pseudoacini, unlike the single cell arrangement in pure myxoid liposarcomas (13,16). Overall, as a result, the distinction between the tumors is occasionally difficult. It is therefore important to conduct long-term follow-up in order to remain alert to the correct diagnosis.

The differential diagnosis for DFML includes other myxoid spindle cell neoplasms of the soft tissue, including superficial angiomyxoma, superficial acral fibromyxoma and myxoid perineuroma (1–6,8). When a myxoid spindle cell tumor is diagnosed, DFML should be considered; it is important to avoid misdiagnosis of more aggressive neoplasms.

In summary, the present study reported a case of an unusual DFML of the shoulder, which to our knowledge, was found in the youngest patient presented in the literature to date. This case was unusual in its location and size, and was diagnosed based on the typical morphological criteria of DFML and the exclusion of malignant features. Further studies with more cases will be required to determine whether the tumor belongs to a myxoid variant of SCLs, an unusual form of myxoid SFT or an intermediate type between the two.
